# Molecular Link Between Psoriasis and Depression—Update on Pathophysiology

**DOI:** 10.3390/ijms26062467

**Published:** 2025-03-10

**Authors:** Agnieszka Hołdrowicz, Agnieszka Żebrowska

**Affiliations:** Department of Dermatology and Venereology, Medical University of Lodz, 90-647 Lodz, Poland; a.holdrowicz@wp.pl

**Keywords:** psoriasis, depression, pathogenesis

## Abstract

Psoriasis disease is a chronic, systemic condition. Various epidemiological studies have indicated a connection between psoriasis and psychiatric diseases. It is obvious that easily visible psoriatic skin lesions cause stigmatization of patients and impact noticeably their life quality, increasing the risk of anxiety and depressive disorders. More and more attention is recently being paid to the common pathogenesis of psoriasis and depression. The underlying cause of psoriasis is chronic inflammation, and depression is also increasingly recognized as a result of neuroinflammation. Therefore, the complexity of the processes underlying both disease entities implies the need to observe psoriatic patients in terms of possible comorbidities, such as mental disorders, regardless of the severity of skin lesions and social stigmatization. This study aims to present an update on the common pathophysiology of both diseases.

## 1. Introduction

Psoriasis is a chronic disease that has a substantial impact on the quality of life. Various epidemiological studies have indicated a connection between psoriasis and psychiatric diseases, such as depression, anxiety disorders [1,2], obsessive-compulsive disorders [3], schizophrenia [4], or dementia [5]. Among the factors increasing the risk of depressive disorders in adolescent and adult patients with psoriasis are joint inflammations. Moreover, a delay in the diagnosis of psoriatic arthritis of more than 6 months increases the risk of depression [6,7]. Both joint involvement and skin symptoms profoundly affect life quality and exacerbate anxiety and depression [8].

Female gender, sedentary lifestyle, sleep disorders, and pain are additional factors aggravating the risk of anxiety and depressive disorders. The co-occurrence of obesity and older age is also associated with a decreased life quality [9]. On the other hand, in children suffering from psoriasis, a higher risk of anxiety, depression, and suicidal thoughts and behaviors was observed [10]. The incidence rate of the latter in adult patients varies depending on the study group but seems to be similar to the general population [11,12,13]. The connection between the severity of skin lesions in the course of psoriasis and the risk of depressive disorders is also unclear and requires further studies [11,12,14]. In a population-based study, a statistically significant relationship between psoriasis and decreased sleep quality was confirmed [15].

It is obvious that easily visible psoriatic skin lesions cause stigmatization of patients and impact noticeably their life quality, increasing the risk of anxiety and depressive disorders. More and more attention is recently being paid to the common pathogenesis of both disease entities. The underlying cause of psoriasis is chronic inflammation; therefore, psoriasis should not be classified as a skin and joint disease, but as a systemic disease. Depression is also increasingly recognized as a result of neuroinflammation. It has been observed that C-reactive protein (CRP) and erythrocyte sedimentation rate (ESR) levels were higher in a group of patients suffering from psoriasis and depression than in psoriatic patients without depression [16].

This study aims to present the common background of both disease entities, considering papers mainly published after 1 January 2022.

## 2. Methods

PubMed and Scopus were searched for the phrases “psoriasis and depression”, “genetics”, “immunology”, “blood-brain barrier”, “skin-brain axis”, “hypothalamic-pituitary-adrenal axis”, “brain abnormalities”, and “mental disease” to perform this non-systematic review. The final search was conducted on 5 January 2025. The main focus was on papers published after 1 January 2022. Older articles were included only if necessary to describe the background in detail. Only full-text articles in English were taken into consideration.

## 3. Genetic Background

In the pathogenesis of psoriasis, the genetic background is of considerable importance. In recent years, numerous studies have been published analyzing the genetic connection between psoriasis and psychiatric diseases. The outcomes of research based on gene sequencing are not unambiguous. It has been assessed using genome-wide association study (GWAS) summary-level data that psoriasis is genetically positively correlated not only with depression but also with heart failure, coronary artery disease, and type 2 diabetes. The Mendelian randomization test has indicated such a connection for coronary artery disease, but it has not been confirmed by sensitivity analyses. It should also be noted that diseases possibly genetically related to psoriasis have common risk factors, including obesity and nicotinism [17].

In the study conducted by Wang et al. [18] using data from the GWAS database and the Mendelian randomization analysis, a potential causal relationship between psoriasis with major depressive disorder and bipolar disorder was determined. However, it should be mentioned that the genetic risk of bipolar disorder was significantly higher than that of depression. No connection between psoriatic arthritis and depression was found [18]. Similar research based on the GWAS database for the European population revealed that from a genetic point of view, psoriasis is not a risk factor for the development of major depression. On the other hand, it seems that bipolar disorder is a risk factor for psoriasis occurrence [19].

The relationship between genetically determined character traits and the possible occurrence of psoriasis was also analyzed. It was proven that genetic susceptibility to psoriasis is associated with a tendency to experience worry [20,21] and other negative emotions [21,22]. Evidence on the connection between psoriasis and neuroticism is inconsistent [20,22]. It should be mentioned that a significant horizontal pleiotropy between psoriasis and depressed affect was observed [20]. Moreover, the occurrence of Single Nucleotide Polymorphism (SNP) predisposing to depression and psoriatic arthritis, localized on the same chromosomal region 6p21.1, was also noticed [23].

Based on an analysis of gene expression from the Gene Expression Omnibus (GEO) database, 115 common differentially expressed genes for psoriasis and depression were found, of which 55 were up-regulated and 60 were down-regulated. Subsequently, a protein–protein interaction (PPI) network was created by selecting 17 hub genes (CTLA4, LCK, ITK, IL7R, CD3D, SOCS1, IL4R, PRKCQ, SOCS3, IL23A, PDGFB, PAG1, TGFA, FGFR1, RELN, ITGB5, and TNXB) from among 115 genes. Most of these are engaged in immunological processes mediated by T cells, which play a pivotal role in the pathogenesis of psoriasis and depression [24].

The Wnt/β-catenin signaling pathway is involved in the modulation of tumor necrosis factor α (TNF-α)-dependent inflammation in keratinocytes. The peroxisome proliferator-activated receptor (PPARγ), participating in the regulation of amino acids, and glucose and lipid metabolisms, and in terminal keratinocyte differentiation, is down-regulated in patients suffering from psoriasis, as well as in patients with depression [25].

Using data from the GEO database, 395 commonly differentially expressed genes for psoriasis and depression were found, and out of these, 20 core genes associated with the Wnt signaling pathway were selected. The significance of the aforementioned core genes in the pathogenesis of psoriasis and depression was confirmed with a mouse model. Both in mice with psoriasis-like skin lesions caused by topically applied imiquimod (IMQ) and in mice with developed symptoms of depression due to stress factors, increased levels of CD19 were noticed. Moreover, the highest CD19 levels were noted in a combined model in which mice were initially subjected to an Unpredictable Mild Stress (CUMS) procedure and then psoriasis-like skin changes were induced by imiquimod. In addition, after applying lentivirus, a topical CD19 inhibitor, on skin lesions, a considerable improvement was observed in the area of psoriatic skin changes. It was confirmed that suppressing CD19 expression increases PPARγ, Wnt3a, and β-catenin levels. Furthermore, lentivirus was applied directly into the prefrontal cortex brain area of mice undergoing CUMS procedure and resulted in a reduction in depression symptoms. As in the case of psoriasis, inhibiting the CD19 expression caused an increase in the concentration of PPARγ, Wnt3a, and β-catenin; therefore, it is probable that CD19 regulates the symptoms of psoriasis and depression through the PPARγ/β-catenin/Wnt3a signaling pathway [25]. In this regard, the CD19 protein can be used not only as a biomarker of psoriasis and depression but also as a potential therapeutic target for new drugs. Moreover, in a group of patients with depression who committed suicide, a changed expression of the CD19 gene was reported [26]. Further studies on the clinical significance of targeting the Wnt/β-catenin signaling pathway in the therapy of psoriasis and depression are needed.

CD19 is also a biomarker for B lymphocytes, and increased levels of these cells were observed not only in the peripheral blood of patients with psoriasis but also in depression [25,27]. In contrast, other studies conducted on patients with depression reported decreased levels of naïve B lymphocytes and memory B cells; therefore, further studies on the involvement of B cells in the pathogenesis of depression are needed [25,28,29].

The literature on the subject provides a case study of a patient who underwent a CD19 chimeric antigen receptor T (CAR-T) cell therapy due to refractory/relapsed diffuse large Bcell lymphoma, which resulted in a remission of psoriatic skin changes [30]. On the other hand, after treatment with anti-CD20 antibodies, the exacerbation of psoriasis was observed [31,32,33]. It could be associated with an increase in the level of Bcell activating factors, which indirectly impact T cells [34] with the loss of B lymphocytes producing interleukin-10 (IL-10) [35].

In the development of numerous disease entities, epigenetics plays a crucial role in influencing the expression of various genes [36]. In research conducted on a study group consisting of students anonymously declaring depression, suicidal thoughts, or self-mutilation and a control group with no mental disorders, the methylation of DNA isolated from saliva was analyzed. There were disparities found in the DNA methylation of *LCE3* genes and *MIR4520A/B* genes involved in the pathogenesis of psoriasis. The Late Cornified Envelope-3 (LCE3) genes localized on chromosome 1 encode the components of stratum corneum and are presumably involved in keratinocytes differentiation. Moreover, the *MIR4520A/B* locus encodes *miR4520A* and *–B*. It was confirmed that, in the area of psoriatic lesions, the *miR4520A* was statistically significantly downregulated. In the study, an increased methylation of the *MIR4520A/B* locus was also observed, which could cause a downregulation of this gene. In addition, a loss of methylation in the *PSORS1C3* gene was confirmed. It should be mentioned that genetic predisposition is not the sole prerequisite for disease development. In the evaluated study group, no students manifested any psoriasis symptoms [37].

## 4. Immunological Background

In the pathogenesis of psoriasis, two lines of T cells, Th1 and Th17 cell lines, and their cytokines are of utmost significance. It is assumed that Th17 cells and IL-17 also participate in the pathogenesis of depression [38]. In research using mouse models, male BALB/c mice were subjected to a topically applied 5% imiquimod cream and developed psoriasiform dermatitis. Mice with skin lesions underwent behavioral tests, confirming the occurrence of depressive disorders [39].

Microgliopathy, i.e., a dysfunction of microglia being a major immune system component in the central nervous system, is also substantially participating in the pathogenesis of depression. On the surface of microglia are located receptors for IL-17A, which stimulates microglia cells into producing pro-inflammatory cytokines such as IL-1β, IL-6, and TNF-α. It is known that microglia stimulation in areas of the hippocampus, prefrontal cortex, amygdala, and anterior cingulate cortex is involved in the development of depression [40,41]. Not only microglia activation in areas of the hippocampus and cortex but also inhibition of hippocampal neurogenesis was observed in mice with psoriasiform dermatitis. In addition, an increased secretion of proinflammatory cytokines (IL-1β, IL-6, and TNF-α) by microglia was noticed [39]. However, microglia, under certain circumstances, also have the ability to produce anti-inflammatory cytokines (IL-4 and IL-10) [41]. In mice with skin lesions, stable levels of these cytokines were noted [39].

Proinflammatory cytokines secreted by microglia may induce the differentiation of CD4^+^ T cells into Th17 cells, leading to the in situ production of IL-17A. Moreover, under pathological conditions, self-production of IL-17 by microglia may occur [39,42]. Mice received anti-IL-17 intraventricularly. Following administration of the drug, a reduction in symptoms of depression was noticed. In addition, the concentration of proinflammatory cytokines was lower, and a decreased microglia activation in areas of the hippocampus and cortex was observed [39].

In the course of neuroinflammation, which underlies depression, the involvement of astrocytes is also observed. There are two different types of astrocyte activation—A1 and A2. The A1-type astrocyte activation is caused by TNF-α and IL-1 cytokines and by a component subunit 1q (C1q) secreted by activated microglia cells. A1-type-activated astrocytes have a neurotoxic effect and may cause the necrosis of neurons. The influence of A1 reactive astrocytes was confirmed in various neurodegenerative diseases [42,43,44]. Supposedly, TNF-α can activate primary astrocytes into A1-type through tumor necrosis factor receptor 1 (TNFR1). The overexpression of TNF-α, TNFR1, chemokine (C-X-C motif) ligand 1(CXCL1), complement C3, and cleaved-caspase-3 in the hippocampus was also observed in mice undergoing the CUMS procedure. Moreover, an increased apoptosis of neurons was reported. TNF-α binding to TNFR1 promotes an inflammatory response, activation of immunological cells, and neurodegenerative processes. It was noticed that the TNFR1 knockdown decreases depressive symptoms [45].

In patients suffering from depression, a changed profile of pro-inflammatory and anti-inflammatory cytokines in peripheral blood in comparison to healthy controls [46] can be also noticed. Patients with both psoriatic arthritis (PsA) and depression had statistically significantly higher levels of IL-1 and IL-6 in their peripheral blood than PsA patients without depression. No such correlation was observed for IL-17, IL-23, and TNF-α [47]. An increased concentration of IL-6 in patients suffering from PsA and depressive disorder compared to the PsA-group was also confirmed in a different research [48].

The pro-inflammatory cytokines TNF-α, IL-17, and IL-6 play a crucial role in both psoriasis and depression by driving chronic inflammation in the skin and neuroinflammation in the central nervous system. TNF-α and IL-6 stimulate each other’s production, amplifying systemic inflammation, while IL-6 promotes Th17 differentiation, leading to increased IL-17 levels. IL-17 and TNF-α contribute to blood–brain barrier dysfunction, facilitating neuroinflammatory responses that may exacerbate depressive symptoms. Additionally, IL-6 activation of the hypothalamic–pituitary–adrenal (HPA) axis results in dysregulated cortisol secretion, affecting neuroplasticity and mood regulation. These findings support the hypothesis that targeting cytokine pathways with biologic therapies may alleviate both psoriatic and depressive symptoms [49].

## 5. Skin–Brain Axis

Due to the co-occurrence of psoriasis and mental illnesses, a skin–brain axis hypothesis has been proposed, which assumes the involvement of the same immunological cells and their cytokines in the pathogenesis of both skin and mental disorders. According to this theory, T cells and pro-inflammatory cytokines can cross the blood–brain barrier (BBB) and impact the sympathetic nervous system and the hypothalamic–pituitary–adrenal axis. One of the most important T lymphocytes participating in the pathogenesis of psoriasis is the Th17 cells secreting IL-17 and IL-22. Interleukin 17 stimulates keratinocytes to produce various chemokines, inducing the influx of neutrophils, lymphocytes, and antigen-presenting cells (APCs) into the skin [50].

In addition, keratinocytes with immune system cells produce pro-inflammatory cytokines, such as, inter alia, TNF-α, IL-1β, and IL-6, stimulating dendritic cells to IL-23 secretion, which increases survival and proliferation of cells releasing IL-17 [50]. In research conducted on laboratory rats, substantially increased levels of IL-1β, IL-6, and TNF-α in serum and brain tissues were reported due to chronic restraint stress [51]. According to Tong et al., statistically significantly higher levels of TNF-α, IL-17A, and IL-23 were observed in patients suffering from both psoriasis and depression compared to psoriatic patients with no depression [52].

**Figure 1:**
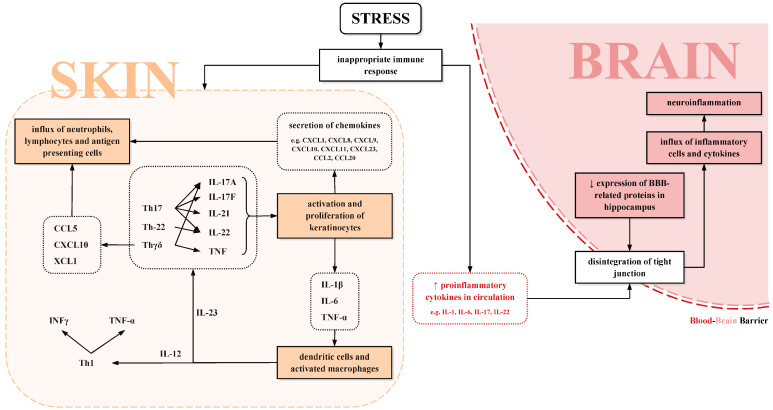
The impact of stress on the skin–brain axis [50,53,56,57].

An important factor playing a vital role in the pathogenesis of psoriasis and depression is stress. It was observed in animal models that the permeability of the blood–brain barrier increases due to chronic stress [51,53]. Chronic stress is supposed to increase levels of pro-inflammatory cytokines in blood circulation, leading to the disintegration of tight junctions between endothelial cells and causing damage to the blood–brain barrier. As a result, the influx of inflammatory cells and cytokines into the brain follows, exacerbating the neuroinflammation [51]. It was also noticed that CUMS-induced depressed mice have in the hippocampus a decreased expression of genes for BBB-related proteins and angiogenesis. Lower levels of proteins, such as Claudin5 and ZO-1, associated with tight junctions were reported [53]. The impact of stress on the skin–brain axis is shown in Figure 1.

Chronic restraint stress causes an increase in the number of Th17 cells and their cytokines in the dorsal striatum, leading to further damage of the BBB, which was confirmed in a rat model study. Moreover, it was proven that inhibiting the differentiation of naïve CD4^+^ T cells into effector Th17 cells beyond the central nervous system (CNS) relieves depressive symptoms induced by exposure to stress. Statistically significantly lower levels of cytokines IL-6, IL-17, and IL-22 in serum were observed; however, IL-17 concentration in dorsal striatum only slightly decreased and IL-22 levels remained stable. This is probably due to the fact that under stress, IL-17 is being produced in CNS and also by some other cells, e.g., gamma delta T cells (γδ T cells) [51]. The presence of these cells was confirmed in the skin of patients suffering from psoriasis and is an important factor in the occurrence of IMQ-induced psoriasiform dermatitis [54].

Gamma delta T cells are T cells with γδ T cell receptors (TCRs) located on their surface. The functions of γδ T cells in the skin and brain are different due to the nature of these tissues and the high heterogeneity of γδ T cells itself. Under the influence of various cytokines, γδ T cells have the capacity to produce either IFN-γ or IL-17A. Moreover, γδ T cells participate in the development of neuroinflammation, especially in the early stage of inflammation. IL-17A secreted by these cells is not only involved in neuroinflammation but also regulates many brain functions. In addition, γδ T cells are engaged in anticancer immunity and in maintaining homeostasis in CNS [55]. They can also infiltrate to the brain parenchyma and participate in the inflammatory response in the course of numerous diseases. In human skin, γδ T cells are primarily found in the dermis and, to a lesser extent, in the epidermis. Dendritic epidermal T cells (DETCs) located in the epidermis are involved in wound healing due to the secretion of various cytokines, chemokines, and growth factors resulting in the inflammatory state and re-epithelialization. Furthermore, γδ T cells participate in antifungal and antiviral response [56].

## 6. Sympathetic Nervous System

The sympathetic nervous system affects cells of the immune system through neuropeptides and neurotransmitters. In the skin, numerous nerve fibers, such as unmyelinated C-fibers, afferent fibers, myelin-type Aδ-fibers, and autonomic nerve fibers, are present. Moreover, on the surface of immune system cells, various receptors, such as, inter alia, epinephrine, acetylcholine, serotonin, norepinephrine, serotonin, substance P, vasoactive intestinal peptides, and endorphins, are located. Neuropeptides secreted by nerve endings can activate and sustain inflammatory conditions in the skin [50,58]. There is evidence from the literature, as given below, regarding the relationship between various neurotransmitter systems and inflammation in psoriatic skin.

The calcitonin gene-related peptide (CGRP) is a neuropeptide involved in sympathetic activity regulation, vasodilation regulation, and neurogenic inflammation. In the area of psoriatic lesions, an increased amount of nerve fibers secreting CGRP is reported. Substance P (SP) expression is observed on various cells, such as astrocytes, microglia cells, neurons, endothelial cells, epithelial cells, macrophages, eosinophils, and dendritic cells. It is also secreted by C and Aδ sensory nerve fibers. SP is involved in the transmission of itch and pain sensations to the CNS [59].

Substance P and the calcitonin gene-related peptide have an ability to induce activation and degranulation of mast cells, leading to the release of pro-inflammatory cytokines and vasoactive amines [60]. In addition, on endothelial cells, neurokinin 1 receptors (NK1Rs) for SP, which induces an increased expression of vascular cell adhesion molecule 1 (VCAM-1) and Intercellular Adhesion Molecule 1 (ICAM-1), are located. Substance P also causes angiectasis, keratinocytes and fibroblasts proliferation, and activates B lymphocytes. An increased level of SP in psoriatic skin lesions is observed. It activates and stimulates the maturation of dendritic cells, which subsequently release IL-12 and IL-23. CGRP stimulates the dendritic cells to secrete IL-23 and may also trigger γδ T cells to produce an inflammatory response [59].On the other hand, the nerve growth factor secreted by keratinocytes and fibroblasts regulates, inter alia, the release of neuropeptides and the proliferation and growth of nerve cells [61]. Norepinephrine induces endothelial cells to produce IL-6, which stimulates the differentiation of T cells into Th17 cells [50,58].

It was confirmed that the dopamine concentration in the blood of patients with psoriasis and depressive disorders was lower compared to psoriatic patients with no psychiatric disorders and to the control group. Furthermore, the dopamine level in this group was negatively correlated with the mean Hamilton Depression Rating Scale (HAMD) score, Athens Insomnia Scale (AIS) score, and Psoriasis Area and Severity Index (PASI) score. No statistically significant differences in the concentration of epinephrine and norepinephrine were observed between the groups [62].

Keratinocytes have also a capacity to produce neurotransmitters, such as, e.g., adrenaline, noradrenaline, histamine, acetylcholine, and dopamine. Additionally, receptors for neurotrophins and neuropeptides are located on their surface [50]. It was confirmed that the α7 subunit of the nicotinic acetylcholine receptor (α7nAChR) is found on both keratinocytes and macrophages in skin, and its expression is higher in the area of psoriatic lesions than in healthy skin. Moreover, activation of this receptor resulted in the remission of the IMQ-induced murine psoriasis model due to suppressing STAT3 and NF-κB signaling pathways [58].

The differentiation of T cells into Th17 cells also occurs under the effect of norepinephrine, which receptors are present on T cells. Adrenoceptor β1 is located on γδT cells and is a primary norepinephrine receptor in the skin. Its stimulation leads to the secretion of IL-17. It was confirmed that cutaneous calcium/calmodulin-dependent protein kinase II-γ (CAMK2γ), presented mostly on sympathetic nerves, is expressed under stress and as a result of topically applied imiquimod. Activated CaMK2γ stimulates the skin sympathetic nerves to norepinephrin secretion, which affects γδT cells through the ARβ1 receptor causing activation of ARβ1–NF-κB and –p38 axes, resulting in IL-17 production. In mice lacking the gene responsible for the encoding of this protein, less severe skin lesions caused by locally applied imiquimod were observed [63]. Furthermore, it was confirmed that the transient receptor potential vanilloid 1 (TRPV1), located on neurons in the skin, may induce a Th17-dependent response and stimulate the proliferation of keratinocytes [64].

## 7. Hypothalamic–Pituitary–Adrenal Axis (HPA Axis)

The role of the hypothalamic–pituitary–adrenal axis in a crosstalk between skin and brain is significant and is increasingly called a neuro–immuno–endocrine–cutaneous axis. The hypothalamus secretes corticotropin-releasing hormone (CRH) causing the production of adrenocorticotropic hormone (ACTH) by the pituitary gland, which stimulates corticosteroid synthesis in the cortex of the adrenal gland [60].The neuroinflammation hypothesis in the pathogenesis of depression assumes that, under psychosocial stressors, proinflammatory cytokines and chemokines are released, which mediate an inflammatory state in the CNS. In the course of inflammatory diseases, a steroid resistance develops, which may be responsible for an HPA axis dysfunction. Proinflammatory cytokines suppress the expression and functioning of the glucocorticoid receptor (GR). This assumption is supported by the fact that biomarkers of inflammation and steroid resistance are simultaneously markers of resistance to depression treatment. It is also considered that a decreased expression of GR mRNA in macrophages may occur due to stress factors [65].

The CRH, ACTH, and glucocorticoids are also produced in the skin due to the pro-inflammatory cytokines, and this phenomenon is named a peripheral HPA axis. CRH is released in the skin by various cells, such as sebocytes, melanocytes, fibroblasts, mast cells, and keratinocytes [66]. It stimulates the expression of pro-inflammatory cytokines in keratinocytes by binding to CRH1-type receptors (CRHR1s) located on their surface [50].

Moreover, the expression of CRHR1 was observed on mast cells. After binding to its receptors, CRH causes the degranulation of mast cells, leading to the release of IL-6, IL-8, IL-22, and vascular endothelial growth factor (VEGF) [66,67]. Pro-inflammatory cytokines like IL-1, IL-6, TNF-α, and IFN-α increase the secretion of CRH, ACTH, and cortisol. Moreover, they decrease the expression and activity of glucocorticoid receptors causing a further increase in the release of cortisol. As a result of CRH binding to its receptor CRHR1, the production of proopiomelanocortin (POMC), at first, and then of ACTH, MSH, and β-endorphin in the skin takes place. ACTH induces the corticosterone secretion by fibroblasts [66]. It was confirmed that in the area of psoriatic skin lesions, the expression of the CRHR1 receptor was higher than in non-lesional skin [67].

In patients with depression, a higher concentration of IL-6 in peripheral blood is observed. It was proven that IL-6 can cross the damaged blood–brain barrier and activate the HPA axis. In the central nervous system, IL-6 can additionally increase the expression of indoleamine dioxygenase (IDO), leading to lower levels of tryptophan (precursor to serotonin) and to an increase in the levels of its catabolites, such as quinolinic acid and kynurenine. The abnormal ratio of these metabolites is an important factor in the pathogenesis of depression [68,69].

Chronic use of opioids also aggravates the risk of depression. In research conducted on rats, long-term oxycodone therapy resulted in an increase in IL-17A and IL-6 levels in the ventral tegmental area (VTA), the development of depressive symptoms, and physical dependence. It was confirmed that anti-IL-17A antibodies prevent the development of depression and decrease the severity of somatic symptoms after treatment withdrawal. The administration of anti-IL-17A caused a normalization of the IL-6 level and a reduction in the concentration of IL-17 in VTA [70].

In a group of patients suffering from depression, increased levels of cortisol, CRH, and ACTH were observed. An increased cortisol level is presumably a consequence of an inflammatory state and contributes to the inflammation participating in the pathogenesis of depression [71].

On the other hand, increased IL-17 and ACTH levels and a lower cortisol concentration, compared to healthy controls, were noticed in patients suffering from psoriasis. Moreover, in psoriatic patients, an increased level of stress evaluated with the Presumptive Stressful Life Events scale (PSLE), Perceived Stress Scale (PSS), and Daily Hassles and Uplifts Scale (DHUS) was observed. Patients with measured high stress-levels had lower cortisol concentrations in the blood than patients with low or moderate stress levels. It suggests a severe disruption of the HPA axis [72]. Lower cortisol levels in the saliva of psoriatic patients in comparison to the control group were also observed in other studies [73,74]. However, one research conducted on a group of psoriatic patients exposed to acute stress reported increased cortisol levels [75]. It should also be mentioned that, in patients suffering from psoriasis, a local cortisol synthesis in the skin is disturbed. In addition, the expression of GR on keratinocytes in the skin is decreased or GR malfunction is observed [76]. Further research is needed to be conducted to determine the role of the HPA axis in the pathogenesis of psoriasis and depression.

## 8. Brain Function and Structure Abnormalities

Much research evaluating brain abnormalities in both patients with psoriasis and depression or on mice models has been conducted in recent years.

Chen et al. [77] noticed that, in C57-BL6 mice models with IMQ-induced dermatitis, anxiety symptoms occurred. Moreover, individuals presenting such behavior tended to isolate themselves from other animals. In a group of mice with skin lesions and using an 11.7T resting-state functional magnetic resonance imaging, changes in the volume of gray matter in the basal forebrain region, anterior commissure intrabulbar, and striatum compared to controls were observed. The aforementioned brain regions are associated with a feeling of anxiety. In addition, there was an observed increased connectivity between various brain areas and the amygdala [77].

The hyperactivation of the amygdala plays a crucial role in both depressive and anxiety disorders. In a healthy individual, the amygdala is inhibited, thus counteracting inappropriate emotions and behaviors caused by a minor stimulus. Stress conditions cause an excessive activation of this brain part, resulting in a tendency for overreactions induced by even a minor external stimulus. In addition, the amygdala is an activator of the HPA axis. In mice models exposed to chronic unpredictable stress, an increased expression of cytokines IL-1β, IL-6, and TNF-α in the amygdala was observed and mice presented symptoms of depression and anxiety disorder. Moreover, in rats subjected to chronic restraint stress, an increased expression of NLRP3 and IL-1β in the basolateral amygdala region was reported. Similarly, in patients exposed to stressors, elevated levels of proinflammatory cytokines and increased amygdala activation were observed [78].

Lada et al. [79] conducted research assessing magnetic resonance brain images (MRI) of patients with psoriasis and depression. An increased thickness of the right precuneus cortex was observed in patients with both psoriasis and depression in comparison to a group suffering from a depressive disorder with no psoriasis comorbidity. However, no significant differences were noticed in subcortical or cortical volumes, surface area, and white matter integrity in psoriatic patients compared to controls without psoriasis, regardless of the co-occurrence of depression. The mechanism underlying an increased thickness of the precuneus has not yet been fully determined. No correlation between the described phenomenon and inflammation markers was observed. However, neuroinflammation involvement should not be excluded since only the amount of neutrophils and CRP levels in peripheral blood were assessed. Changes observed in the brain structure may also correspond to alterations in somatosensory processing and be related to recurrent lifetime suicidality. In patients suffering from psoriasis with co-existing PsA, a reduced resting-state connectivity between the superior frontal and temporo-occipital cortices was observed in comparison to patients with no joint involvement and to non-psoriatic individuals. The observed changes can be associated with a feeling of chronic pain and a systemic inflammatory condition [79].

Wang et al. [80] used resting-state functional magnetic resonance imaging (fMRI) scanning to evaluate the fractional amplitude of low-frequency fluctuations (fALFFs) and functional connectivity (FC) in a group of patients with psoriasis and depression. The control group consisted of healthy volunteers. In the study group, increased fALFF values in the left middle frontal gyrus (Frontal_Mid_L) and higher positive FC between the right hypothalamus and the right median cingulate (Cingulum_Mid_R) were observed. Moreover, negative correlations between fALFF and FC values, and also between self-rating depression scale (SDS) and psoriasis area and severity index (PASI) scores were reported [80]. In numerous studies conducted on patients with depression, abnormal brain activity in areas of the hypothalamus and middle frontal gyrus was noticed [81,82,83]. It was also confirmed that in patients suffering from treatment-refractory depression, the volume of gray matter in both the middle frontal gyrus and anterior cingulate cortex is reduced [81].

Marek-Jozefowicz et al. [84] confirmed that patients with psoriasis achieved neuropsychological test results, suggesting worse cognitive abilities in comparison to healthy controls of similar age and with comparable education levels. No relationship between the occurrence of cognitive dysfunction and the severity of depression on the Hospital Anxiety and Depression Scale (HADS) was determined. However, such a correlation was confirmed for psychomotor slowness. In MRI scans of psoriatic patients, only low or moderate amounts of white matter lesions were found, but changes in the brain were generally more extensive compared to the control group. It has been confirmed that the less severe lesions in the white matter, the higher brain volume and gray matter volume are, the better cognitive function [84].

Patients with psoriasis more often suffer from metabolic syndrome [85,86] and are, due to this fact, more susceptible to vascular-related brain degeneration affecting cognitive functions. Dadkhahfar et al. [87] reported no differences in the frequency of vessel cerebrovascular disease between patients with psoriasis and healthy controls. However, a correlation between cerebral atrophy and the disease duration was found [87].

The association between inflammatory cytokines and cognitive test scores in psoriatic patients has not been determined yet. However, in a group of older patients, an increased TNF-α level was related to a worse immediate memory learning. Such a correlation was statistically significant, even after adjusting for age [88]. It was also noticed that raised inflammatory markers in a group of patients suffering from rheumatoid arthritis are associated with a higher risk of cognitive impairment [89].Taking into consideration the anti-inflammatory effect of drugs used in psoriasis treatment, there is a need to conduct prospective studies evaluating the influence of psoriasis therapy on depressive symptoms and long-term cognitive outcomes.

## 9. Therapy

Taking into account similar inflammatory background and participation of the same cytokines in the pathogenesis of psoriasis and depression, it seems potentially possible to use these common points in the therapy of both disease entities.

It has been confirmed in a systematic review comprising 56 clinical studies that almost all drugs used in the therapy of psoriasis have a positive influence on the mental health of patients, reducing symptoms of depression. Biological drugs are considered to yield the best results. Research findings in the case of infliximab are inconsistent. It seems that infliximab may decrease depressive symptoms, but only in patients with a high inflammatory state. Among various drugs used in the therapy of depression, escitalopram, topiramate, moclobemide, gabapentin, and paroxetine seem to be most beneficial in terms of psoriasis comorbidity. On the other hand, lithium and benzodiazepines can aggravate psoriatic skin lesions. Drugs belonging to the Selective Serotonin Reuptake Inhibitors (SSRIs) group have an immunomodulatory effect, which would explain their effectiveness also in the treatment of psoriasis [90].

On the other hand, another study revealed that in a group of patients treated formerly with antidepressants, psoriasis was more frequently observed than in a control group; therefore, the psoriasis-inducing effect of these drugs cannot be excluded [91]. However, it is difficult to eliminate the influence of depression, anxiety, and chronic stress, which are common concomitant factors of psoriasis, on research results. The literature on the subject provides reports of the protective effect of antidepressants, especially drugs belonging to the SSRIs group, on the onset and course of psoriasis [92,93]. However, lithium is regarded explicitly to have a negative impact on the course of psoriasis [91,93]. More studies on this subject are needed.

Wang et al. [90] using published research data calculated Cohen’s d value and confirmed that secukinumab yields the best results in terms of anxiety reduction measured with the Hospital Anxiety and Depression Scal—Anxiety (HADS-A) rating scale. Moreover, ixekizumab was reported to best relieve depressive symptoms assessed with a 16-item Quick Inventory of Depressive Symptomology—Self-Report (QIDS-SR16) index [90].

It was also determined that guselkumab has low effectiveness in the reduction inanxiety (Cohen’s d ≥ 0.2) and moderate in alleviating depressive symptoms (Cohen’s d ≥ 0.5). On the other hand, adalimumab demonstrated medium and high effectiveness, respectively [90]. However, the analysis performed on data from two phase-three clinical trials VOYAGE 1 and VOYAGE 2 indicated that guselkumab has a more substantial influence on the dermatology-specific health-related quality of life (HRQoL) compared to adalimumab, even after excluding the clinical impact on skin changes. In addition, patients undergoing guselkumab therapy in comparison to patients treated with adalimumab reported a more significant decrease in the severity of anxiety and the alleviation of depressive symptoms [94].

Tildrakizumab was not involved in the abovementioned analysis. However, Travoto et al. [95] analyzed the quality of life of patients treated with two different tildrakizumab doses. Both groups were of comparable demographic characteristics. The life quality was assessed using Dermatology Life Quality Index (DLQI) and The World Health Organization-Five Well-Being Index (WHO-5) rating scales after 4, 16, and 28 weeks of therapy. Patients receiving higher regimens of tildrakizumab reported faster and more significant improvement in both scales [95].

The positive influence of biological drugs on the mental health of patients was also confirmed in a retrospective study conducted on US Administrative Claims Data from the MerativeMarketScan^®^ database. It was observed that patients undergoing biological therapies had a 17% lower risk of depression than patients treated with conventional synthetic disease-modifying drugs [96]. It seems that monoclonal antibodies used in psoriasis treatment could also be effective in depression therapy, but this hypothesis requires further studies [97].

Researchers reported not only the association of psoriasis with depression, anxiety, and schizophrenia but also with sexual dysfunction [98,99]. In a 6-month-long observation, a positive influence of adalimumab on erectile function evaluated with the International Index of Erectile Function (IIEF-5) scale was confirmed. In addition, an improvement in sperm motility and vitality and an increase in testosterone concentration were observed [100].

It is not possible to ignore the positive influence of the remission of psoriatic skin lesions on depressive disorder itself; however, research conducted on patients treated with guselkumab [94] confirmed a certain effectiveness of the therapy even after excluding the clinical impact on skin changes. Undoubtedly, there is a need to conduct further prospective studies not only evaluating biological therapies in terms of reducing depression and anxiety disorder risk but also assessing the effectiveness of psoriatic treatment in the therapy of depression.

## 10. Conclusions

Despite the fact that the association between psoriasis and mental disorders has not been completely determined yet, this review focuses on recent studies exploring the common mechanisms underlying both depression and psoriatic disease. In the pathogenesis of psoriasis and depression participate shared factors due to the involvement of the neuro–immuno–endocrine–cutaneous axis. The complexity of the processes underlying both disease entities implies the need to observe psoriatic patients in terms of possible comorbidities, such as mental disorders, regardless of the severity of skin lesions and social stigmatization. This is a highly relevant topic, as mental health disorders can be assessed and effectively managed. Therefore, an increased awareness of this issue in the psoriatic population is crucial. It is also necessary to conduct further studies evaluating both the influence of drugs used in the therapy of psoriasis on the mental health of patients and the impact of antidepressants on the severity of skin lesions in the course of psoriasis.
